# Poly[[tetra­aqua­tetra­kis­[μ_3_-5-(pyridine-4-carboxamido)­isophthalato]­cobalt(II)­diholmium(III)] tetra­hydrate]

**DOI:** 10.1107/S1600536811042814

**Published:** 2011-10-22

**Authors:** Yi-Fang Deng, Man-Sheng Chen, Chun-Hua Zhang, Xue Nie

**Affiliations:** a Key Laboratory of Functional Organometallic Materials, Hengyang Normal University, Department of Chemistry and Materials Science, Hengyang, Hunan 421008, People’s Republic of China

## Abstract

In the centrosymmetric polymeric title compound, {[CoHo_2_(C_14_H_8_N_2_O_5_)_4_(H_2_O)_4_]·4H_2_O}_*n*_, the Ho^III^ ion is coordinated by one water mol­ecule and four 5-(pyridine-4-carboxamido)­isophthalate (*L*) ligands in a distorted square-anti­prismatic arrangement. The Co^II^ ion, located on an inversion center, is coordinated by two pyridine N atoms, two carboxyl­ate O atoms and two water mol­ecules in a distorted octa­hedral geometry. One *L* ligand bridges two Ho ions and one Co ion through two carboxyl­ate groups and one pyridine N atom. The other *L* ligand bridges two Ho ions and one Co ion through two carboxyl­ate groups, while the uncoordinated pyridine N atom accepts a hydrogen bond from an adjacent coordinated water mol­ecule. Extensive O—H⋯O, N—H⋯O and O—H⋯N hydrogen bonding is present in the crystal.

## Related literature

For related hetero-metallic complexes, see: Chen *et al.* (2011 ▶); Deng *et al.* (2011 ▶); Gu & Xue (2006 ▶); Liang *et al.* (2000 ▶); Prasad *et al.* (2007 ▶); Zhao *et al.* (2003 ▶, 2004 ▶).
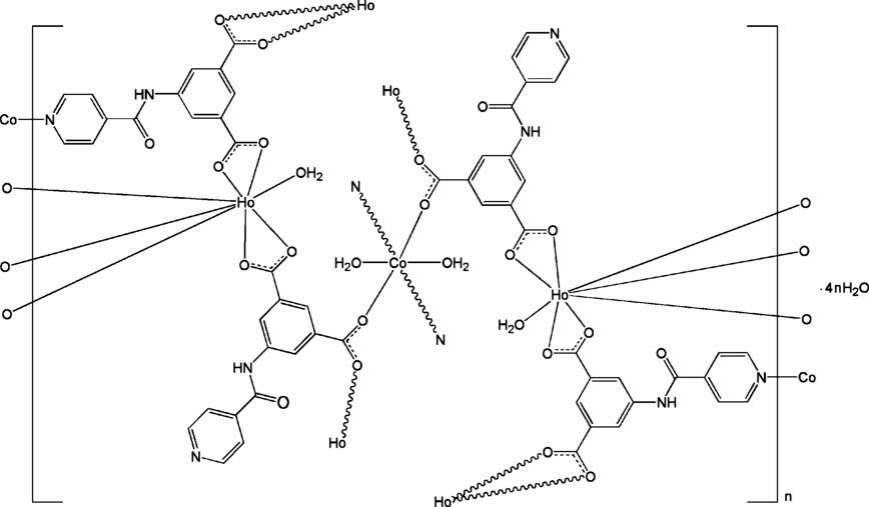


## Experimental

### 

#### Crystal data

[CoHo_2_(C_14_H_8_N_2_O_5_)_4_(H_2_O)_4_]·4H_2_O
*M* *_r_* = 1669.81
Triclinic, 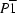

*a* = 10.0597 (9) Å
*b* = 10.7824 (10) Å
*c* = 13.7261 (13) Å
α = 79.141 (3)°
β = 78.801 (1)°
γ = 86.578 (3)°
*V* = 1433.9 (2) Å^3^
*Z* = 1
Mo *K*α radiation
μ = 3.12 mm^−1^
*T* = 291 K
0.20 × 0.14 × 0.10 mm

#### Data collection

Bruker APEX CCD diffractometer
Absorption correction: multi-scan (*SADABS*; Bruker, 2001 ▶) *T*_min_ = 0.574, *T*_max_ = 0.746
7154 measured reflections
4968 independent reflections
4756 reflections with *I* > 2σ(*I*)
*R*_int_ = 0.070

#### Refinement

*R*[*F* ^2^ > 2σ(*F* ^2^)] = 0.046
*wR*(*F* ^2^) = 0.123
*S* = 1.06
4968 reflections
430 parameters
H-atom parameters constrained
Δρ_max_ = 2.89 e Å^−3^
Δρ_min_ = −1.93 e Å^−3^

### 

Data collection: *SMART* (Bruker, 2007 ▶); cell refinement: *SAINT* (Bruker, 2007 ▶); data reduction: *SAINT*; program(s) used to solve structure: *SHELXTL* (Sheldrick, 2008 ▶); program(s) used to refine structure: *SHELXTL*; molecular graphics: *XP* in *SHELXTL*, and *DIAMOND* (Brandenburg, 1999 ▶); software used to prepare material for publication: *SHELXTL*.

## Supplementary Material

Crystal structure: contains datablock(s) global, I. DOI: 10.1107/S1600536811042814/hy2478sup1.cif

Structure factors: contains datablock(s) I. DOI: 10.1107/S1600536811042814/hy2478Isup2.hkl

Additional supplementary materials:  crystallographic information; 3D view; checkCIF report

## Figures and Tables

**Table 1:** Hydrogen-bond geometry (Å, °)

*D*—H⋯*A*	*D*—H	H⋯*A*	*D*⋯*A*	*D*—H⋯*A*
O1*W*—H1*WA*⋯O4*W*^i^	0.85	2.00	2.761 (8)	149
O1*W*—H1*WB*⋯O7^ii^	0.85	2.26	2.988 (7)	144
O2*W*—H2*WB*⋯N2^iii^	0.85	1.96	2.699 (8)	145
O3*W*—H3*WA*⋯O6^iv^	0.85	2.20	3.048 (8)	177
O3*W*—H3*WB*⋯O9^v^	0.85	2.20	3.054 (8)	177
O4*W*—H4*WA*⋯O4^i^	0.85	1.90	2.732 (8)	164
O4*W*—H4*WB*⋯O8^vi^	0.85	1.94	2.752 (8)	160
N1—H1⋯O3*W*	0.86	2.16	2.996 (7)	165
N3—H3⋯O3^vii^	0.86	2.16	2.933 (7)	150

Symmetry codes: (i) 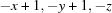; (ii) 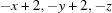; (iii) 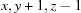; (iv) 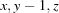; (v) 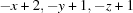; (vi) 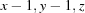; (vii) 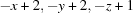.

**Fig. 1.**
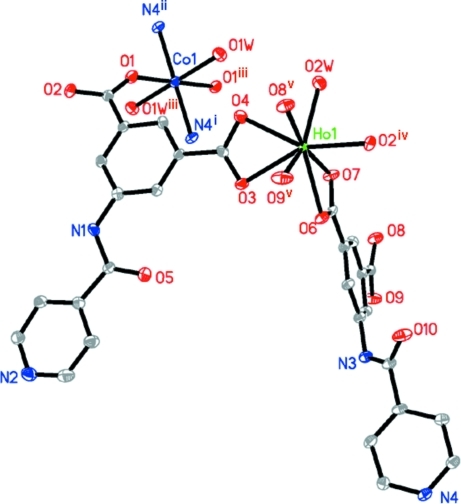
The asymmetric unit of the title compound. Displacement ellipsoids are drawn at the 30% probability level. [Symmetry codes: (i) 2-x, 2-y, 1-z; (ii) x, -1+y, -1+z; (iii) 2-x, 1-y, -z; (iv) x, 1+y, z; (v) -1+x, y, z.]

**Fig. 2.**
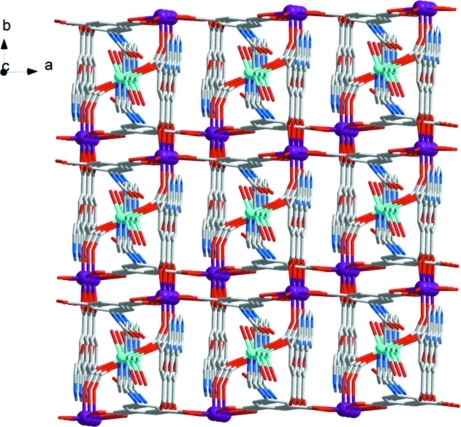
A view of the three-dimensional network of the title compound.

## supplementary crystallographic information

### Comment

The rational synthesis and investigation of 3d-4f or 4d-4f heterometallic complexes are challenge for chemists and have attracted increasing attention in last few years since the competitive reaction containing 3d-4f metal ions in conjunction with ligands often results in the formation of a mixture of homometallic assemblies rather than heterometallic analogous (Gu & Xue, 2006; Liang *et al.*, 2000; Prasad *et al.*, 2007; Zhao *et al.*, 2003, 2004). We have recently prepared a new lanthanide(III)–transition metal(II) coordination polymer, the title compound, under hydrothermal conditions.

In the title compound, the Ho^III^ ion is eight-coordinated by seven O atoms from four pyridine-4-carboxamidoisophthalate (*L*) ligands and one water molecule, forming a distorted square-antiprismatic geometry (Fig. 1). It is interesting that the carboxylate groups of two unique *L* ligands exhibit different coordination modes: one coordinates to two Ho^III^ ions and one Co^II^ ion using its two carboxylate groups with µ_1_-η^1^:η^1^-chelate and µ_2_-η^1^:η^1^-bis-monodentate coordination modes while the pyridyl group is free of coordination, the other one coordinates to two Ho^III^ ions through the carboxylate groups with µ_1_-η^1^:η^1^-chelate coordination mode and to one Co^II^ ion through the pyridyl group. Based on the coordination modes of the carboxylate and pyridyl groups, a complicated three-dimensional network is formed (Fig. 2), which is similar to the complex {[LnCo_0.5_(INAIP)_2_(H_2_O)_2_].2H_2_O}_n_ (Chen *et al.* 2011) and a Gd analogue (Deng *et al.*, 2011).

### Experimental

A mixture of Ho(NO_3_)_3_.6H_2_O (22.5 mg, 0.05 mmol), H_2_*L* (28.6 mg, 0.1 mmol), Co(OAc)_2_.4H_2_O (13.1 mg, 0.05 mmol), NaOH (6.0 mg, 0.15 mmol), MeOH (4 ml) and H_2_O (6 ml) was heated in a 16 ml capacity Teflon-lined reaction vessel at 433 K for 4 days. The reaction mixture was cooled to room temperature over a period of 40 h. The product was collected by filtration, washed with H_2_O and air-dried.

### Refinement

H atoms bonded to C and N atoms were placed geometrically and refiined as riding atoms, with C—H = 0.93 and N—H = 0.86 Å and with *U*_iso_(H) = 1.2*U*_eq_(C, N). The water H atoms were found from difference Fourier maps and refined with restraints of O—H = 0.85 Å and with *U*_iso_(H) = 1.2*U*_eq_(O). The highest residual electron density was found at 0.92 Å from Ho1 atom and the deepest hole at 1.06 Å from Ho1 atom.

### Crystal data

**Table:**

[CoHo_2_(C_14_H_8_N_2_O_5_)_4_(H_2_O)_4_]·4H_2_O	*Z* = 1
*M**_r_* = 1669.81	*F*(000) = 825
Triclinic, *P*1	*D*_x_ = 1.934 Mg m^−^^3^
Hall symbol: -P 1	Mo *K*α radiation, λ = 0.71073 Å
*a* = 10.0597 (9) Å	Cell parameters from 5442 reflections
*b* = 10.7824 (10) Å	θ = 2.7–28.1°
*c* = 13.7261 (13) Å	µ = 3.12 mm^−^^1^
α = 79.141 (3)°	*T* = 291 K
β = 78.801 (1)°	Block, pink
γ = 86.578 (3)°	0.20 × 0.14 × 0.10 mm
*V* = 1433.9 (2) Å^3^	

### Data collection

**Table:**

Bruker APEX CCD diffractometer	4968 independent reflections
Radiation source: fine-focus sealed tube	4756 reflections with *I* > 2σ(*I*)
graphite	*R*_int_ = 0.070
φ and ω scans	θ_max_ = 25.0°, θ_min_ = 1.9°
Absorption correction: multi-scan (*SADABS*; Bruker, 2001)	*h* = −11→11
*T*_min_ = 0.574, *T*_max_ = 0.746	*k* = −12→10
7154 measured reflections	*l* = −15→16

### Refinement

**Table:**

Refinement on *F*^2^	Primary atom site location: structure-invariant direct methods
Least-squares matrix: full	Secondary atom site location: difference Fourier map
*R*[*F*^2^ > 2σ(*F*^2^)] = 0.046	Hydrogen site location: inferred from neighbouring sites
*wR*(*F*^2^) = 0.123	H-atom parameters constrained
*S* = 1.06	*w* = 1/[σ^2^(*F*_o_^2^) + (0.0881*P*)^2^] where *P* = (*F*_o_^2^ + 2*F*_c_^2^)/3
4968 reflections	(Δ/σ)_max_ = 0.002
430 parameters	Δρ_max_ = 2.89 e Å^−^^3^
0 restraints	Δρ_min_ = −1.93 e Å^−^^3^

### Fractional atomic coordinates and isotropic or equivalent isotropic displacement parameters (Å^2^)

**Table:**

	*x*	*y*	*z*	*U*_iso_*/*U*_eq_	
Co1	1.0000	0.5000	0.0000	0.0253 (3)	
Ho1	0.68057 (2)	1.07784 (2)	0.204341 (17)	0.01470 (13)	
C1	0.6994 (6)	0.4719 (5)	0.2279 (4)	0.0217 (12)	
C2	0.7035 (6)	0.6032 (6)	0.1993 (5)	0.0236 (13)	
H2	0.7193	0.6407	0.1315	0.028*	
C3	0.6834 (6)	0.6770 (5)	0.2746 (4)	0.0221 (12)	
C4	0.6647 (6)	0.6223 (6)	0.3756 (5)	0.0229 (12)	
H4	0.6529	0.6730	0.4246	0.027*	
C5	0.6636 (6)	0.4913 (6)	0.4041 (5)	0.0233 (12)	
C6	0.6770 (6)	0.4169 (6)	0.3298 (5)	0.0240 (13)	
H6	0.6709	0.3296	0.3485	0.029*	
C7	0.6875 (6)	0.8177 (6)	0.2476 (5)	0.0236 (13)	
C8	0.7308 (6)	0.3913 (6)	0.1481 (5)	0.0228 (13)	
C9	0.6680 (7)	0.4839 (6)	0.5835 (5)	0.0289 (14)	
C10	0.6509 (6)	0.4012 (6)	0.6856 (5)	0.0264 (13)	
C11	0.6448 (8)	0.4618 (7)	0.7689 (5)	0.0363 (16)	
H11	0.6452	0.5494	0.7600	0.044*	
C12	0.6382 (8)	0.3898 (8)	0.8632 (6)	0.0425 (18)	
H12	0.6334	0.4310	0.9176	0.051*	
C13	0.6446 (7)	0.2083 (7)	0.8032 (5)	0.0366 (16)	
H13	0.6453	0.1205	0.8150	0.044*	
C14	0.6503 (7)	0.2708 (7)	0.7046 (5)	0.0325 (15)	
H14	0.6536	0.2260	0.6525	0.039*	
C15	0.9066 (6)	1.1082 (6)	0.2811 (5)	0.0229 (13)	
C16	1.0260 (6)	1.1190 (6)	0.3296 (5)	0.0238 (13)	
C17	1.1593 (6)	1.1035 (6)	0.2809 (5)	0.0248 (13)	
H17	1.1774	1.0870	0.2154	0.030*	
C18	1.2633 (6)	1.1125 (6)	0.3300 (5)	0.0228 (13)	
C19	1.2362 (6)	1.1400 (6)	0.4285 (5)	0.0252 (13)	
H19	1.3070	1.1440	0.4623	0.030*	
C20	1.1051 (6)	1.1606 (6)	0.4744 (4)	0.0231 (12)	
C21	0.9979 (6)	1.1474 (6)	0.4250 (5)	0.0234 (13)	
H21	0.9086	1.1579	0.4568	0.028*	
C22	1.4092 (6)	1.0971 (5)	0.2832 (5)	0.0229 (13)	
C23	0.9939 (6)	1.2942 (6)	0.5906 (5)	0.0257 (13)	
C24	0.9915 (6)	1.3357 (6)	0.6897 (5)	0.0264 (13)	
C25	0.9898 (7)	1.4651 (7)	0.6893 (5)	0.0302 (15)	
H25	0.9920	1.5228	0.6294	0.036*	
C26	0.9847 (7)	1.5063 (6)	0.7794 (5)	0.0294 (14)	
H26	0.9852	1.5929	0.7780	0.035*	
C27	0.9748 (7)	1.3039 (6)	0.8680 (5)	0.0313 (15)	
H27	0.9658	1.2485	0.9295	0.038*	
C28	0.9831 (7)	1.2549 (6)	0.7813 (5)	0.0306 (14)	
H28	0.9831	1.1679	0.7844	0.037*	
N1	0.6462 (5)	0.4309 (5)	0.5055 (4)	0.0259 (11)	
H1	0.6196	0.3541	0.5196	0.031*	
N2	0.6383 (6)	0.2642 (6)	0.8813 (5)	0.0399 (14)	
N3	1.0799 (5)	1.1948 (5)	0.5708 (4)	0.0260 (11)	
H3	1.1190	1.1529	0.6176	0.031*	
N4	0.9792 (5)	1.4296 (5)	0.8675 (4)	0.0269 (12)	
O1	0.8061 (5)	0.4321 (4)	0.0666 (3)	0.0333 (11)	
O2	0.6754 (5)	0.2834 (4)	0.1695 (4)	0.0331 (11)	
O3	0.7064 (6)	0.8823 (4)	0.3105 (4)	0.0398 (13)	
O4	0.6712 (6)	0.8746 (4)	0.1615 (4)	0.0411 (13)	
O5	0.6942 (7)	0.5942 (5)	0.5749 (4)	0.0603 (18)	
O6	0.7902 (4)	1.1165 (4)	0.3309 (3)	0.0270 (9)	
O7	0.9238 (5)	1.0921 (5)	0.1893 (3)	0.0320 (11)	
O8	1.4436 (5)	1.0967 (5)	0.1889 (4)	0.0333 (11)	
O9	1.4993 (5)	1.0839 (5)	0.3355 (3)	0.0405 (12)	
O10	0.9226 (5)	1.3484 (5)	0.5328 (4)	0.0421 (13)	
O1W	0.9246 (5)	0.6839 (4)	−0.0658 (3)	0.0366 (11)	
H1WA	0.8426	0.7036	−0.0440	0.044*	
H1WB	0.9828	0.7414	−0.0764	0.044*	
O2W	0.7208 (5)	1.0991 (5)	0.0330 (3)	0.0399 (12)	
H2WA	0.8039	1.0888	0.0078	0.048*	
H2WB	0.6856	1.1678	0.0062	0.048*	
O3W	0.5839 (8)	0.1598 (5)	0.5164 (4)	0.0640 (19)	
H3WA	0.6415	0.1444	0.4657	0.077*	
H3WB	0.5633	0.0901	0.5562	0.077*	
O4W	0.3038 (7)	0.1634 (8)	0.0325 (5)	0.084 (3)	
H4WA	0.3255	0.1440	−0.0260	0.101*	
H4WB	0.3604	0.1340	0.0701	0.101*	

### Atomic displacement parameters (Å^2^)

**Table:**

	*U*^11^	*U*^22^	*U*^33^	*U*^12^	*U*^13^	*U*^23^
Co1	0.0279 (7)	0.0260 (6)	0.0239 (6)	−0.0029 (5)	−0.0040 (5)	−0.0097 (5)
Ho1	0.01438 (18)	0.01247 (18)	0.01903 (18)	0.00036 (11)	−0.00550 (11)	−0.00507 (11)
C1	0.021 (3)	0.019 (3)	0.027 (3)	0.002 (2)	−0.008 (2)	−0.008 (2)
C2	0.019 (3)	0.022 (3)	0.028 (3)	−0.001 (2)	−0.001 (2)	−0.002 (2)
C3	0.025 (3)	0.018 (3)	0.026 (3)	0.001 (2)	−0.008 (2)	−0.007 (2)
C4	0.020 (3)	0.021 (3)	0.029 (3)	0.002 (2)	−0.006 (2)	−0.006 (2)
C5	0.015 (3)	0.024 (3)	0.029 (3)	0.003 (2)	−0.002 (2)	−0.005 (2)
C6	0.027 (3)	0.018 (3)	0.026 (3)	0.000 (2)	−0.003 (3)	−0.006 (2)
C7	0.024 (3)	0.023 (3)	0.024 (3)	0.006 (2)	−0.007 (2)	−0.002 (3)
C8	0.017 (3)	0.024 (3)	0.029 (3)	0.000 (2)	−0.008 (2)	−0.005 (3)
C9	0.036 (4)	0.026 (3)	0.029 (3)	−0.005 (3)	−0.013 (3)	−0.005 (3)
C10	0.022 (3)	0.028 (3)	0.030 (3)	0.004 (2)	−0.010 (3)	−0.003 (3)
C11	0.044 (4)	0.036 (4)	0.032 (4)	0.003 (3)	−0.008 (3)	−0.011 (3)
C12	0.046 (5)	0.055 (5)	0.030 (4)	0.000 (4)	−0.008 (3)	−0.016 (4)
C13	0.037 (4)	0.037 (4)	0.037 (4)	−0.007 (3)	−0.017 (3)	0.000 (3)
C14	0.032 (4)	0.039 (4)	0.029 (3)	−0.007 (3)	−0.011 (3)	−0.005 (3)
C15	0.018 (3)	0.022 (3)	0.031 (3)	−0.003 (2)	−0.005 (3)	−0.010 (3)
C16	0.020 (3)	0.023 (3)	0.031 (3)	0.000 (2)	−0.008 (3)	−0.008 (3)
C17	0.023 (3)	0.027 (3)	0.025 (3)	0.002 (2)	−0.005 (2)	−0.005 (2)
C18	0.020 (3)	0.022 (3)	0.028 (3)	−0.001 (2)	−0.005 (2)	−0.007 (2)
C19	0.019 (3)	0.030 (3)	0.030 (3)	0.002 (2)	−0.013 (3)	−0.007 (3)
C20	0.023 (3)	0.024 (3)	0.026 (3)	0.005 (2)	−0.010 (2)	−0.011 (2)
C21	0.016 (3)	0.029 (3)	0.028 (3)	−0.001 (2)	−0.006 (2)	−0.010 (3)
C22	0.021 (3)	0.019 (3)	0.028 (3)	0.000 (2)	−0.003 (3)	−0.003 (2)
C23	0.019 (3)	0.034 (3)	0.026 (3)	0.001 (3)	−0.006 (3)	−0.011 (3)
C24	0.018 (3)	0.039 (4)	0.026 (3)	0.003 (3)	−0.007 (2)	−0.015 (3)
C25	0.029 (4)	0.038 (4)	0.025 (3)	0.000 (3)	−0.008 (3)	−0.008 (3)
C26	0.030 (4)	0.029 (3)	0.031 (3)	−0.001 (3)	−0.004 (3)	−0.013 (3)
C27	0.033 (4)	0.030 (4)	0.032 (4)	−0.005 (3)	−0.008 (3)	−0.004 (3)
C28	0.032 (4)	0.030 (3)	0.032 (3)	−0.006 (3)	−0.005 (3)	−0.014 (3)
N1	0.034 (3)	0.018 (3)	0.025 (3)	0.001 (2)	−0.006 (2)	0.001 (2)
N2	0.041 (4)	0.046 (4)	0.033 (3)	−0.002 (3)	−0.011 (3)	−0.002 (3)
N3	0.023 (3)	0.030 (3)	0.026 (3)	0.000 (2)	−0.004 (2)	−0.010 (2)
N4	0.024 (3)	0.032 (3)	0.028 (3)	−0.004 (2)	−0.005 (2)	−0.014 (2)
O1	0.033 (3)	0.039 (3)	0.029 (2)	−0.011 (2)	0.001 (2)	−0.011 (2)
O2	0.044 (3)	0.021 (2)	0.038 (3)	−0.004 (2)	−0.010 (2)	−0.009 (2)
O3	0.073 (4)	0.019 (2)	0.036 (3)	0.003 (2)	−0.031 (3)	−0.008 (2)
O4	0.074 (4)	0.021 (2)	0.032 (3)	0.001 (2)	−0.021 (3)	−0.003 (2)
O5	0.113 (6)	0.034 (3)	0.040 (3)	−0.025 (3)	−0.026 (3)	−0.001 (2)
O6	0.018 (2)	0.035 (2)	0.033 (2)	−0.0004 (18)	−0.0057 (18)	−0.0164 (19)
O7	0.026 (2)	0.048 (3)	0.027 (2)	−0.002 (2)	−0.0062 (19)	−0.020 (2)
O8	0.022 (2)	0.049 (3)	0.033 (3)	0.003 (2)	−0.007 (2)	−0.019 (2)
O9	0.018 (2)	0.074 (4)	0.032 (3)	0.006 (2)	−0.009 (2)	−0.013 (2)
O10	0.040 (3)	0.054 (3)	0.043 (3)	0.019 (2)	−0.022 (2)	−0.027 (2)
O1W	0.038 (3)	0.034 (3)	0.036 (3)	0.005 (2)	−0.005 (2)	−0.004 (2)
O2W	0.053 (3)	0.034 (3)	0.031 (3)	0.002 (2)	−0.008 (2)	−0.001 (2)
O3W	0.120 (6)	0.037 (3)	0.035 (3)	−0.023 (3)	−0.009 (3)	−0.008 (2)
O4W	0.059 (4)	0.155 (8)	0.039 (3)	0.029 (4)	−0.018 (3)	−0.022 (4)

### Geometric parameters (Å, °)

**Table:**

Co1—O1	2.098 (4)	C13—H13	0.9300
Co1—O1^i^	2.098 (4)	C14—H14	0.9300
Co1—N4^ii^	2.148 (5)	C15—O6	1.243 (7)
Co1—N4^iii^	2.148 (5)	C15—O7	1.282 (7)
Co1—O1W^i^	2.176 (5)	C15—C16	1.502 (8)
Co1—O1W	2.176 (5)	C16—C21	1.373 (8)
Ho1—O2^iv^	2.177 (4)	C16—C17	1.393 (9)
Ho1—O2W	2.278 (5)	C17—C18	1.367 (9)
Ho1—O9^v^	2.307 (5)	C17—H17	0.9300
Ho1—O6	2.341 (4)	C18—C19	1.411 (8)
Ho1—O3	2.358 (4)	C18—C22	1.496 (8)
Ho1—O4	2.385 (4)	C19—C20	1.371 (9)
Ho1—O8^v^	2.427 (5)	C19—H19	0.9300
Ho1—O7	2.426 (5)	C20—C21	1.408 (8)
Ho1—C22^v^	2.745 (6)	C20—N3	1.412 (7)
C1—C6	1.393 (8)	C21—H21	0.9300
C1—C2	1.397 (8)	C22—O9	1.248 (7)
C1—C8	1.499 (8)	C22—O8	1.274 (8)
C2—C3	1.397 (8)	C23—O10	1.217 (8)
C2—H2	0.9300	C23—N3	1.370 (8)
C3—C4	1.381 (9)	C23—C24	1.506 (8)
C3—C7	1.494 (8)	C24—C28	1.379 (9)
C4—C5	1.393 (8)	C24—C25	1.394 (10)
C4—H4	0.9300	C25—C26	1.382 (9)
C5—C6	1.394 (8)	C25—H25	0.9300
C5—N1	1.403 (8)	C26—N4	1.324 (9)
C6—H6	0.9300	C26—H26	0.9300
C7—O3	1.254 (8)	C27—N4	1.358 (9)
C7—O4	1.261 (8)	C27—C28	1.377 (9)
C8—O1	1.242 (7)	C27—H27	0.9300
C8—O2	1.279 (7)	C28—H28	0.9300
C9—O5	1.212 (8)	N1—H1	0.8600
C9—N1	1.363 (8)	N3—H3	0.8600
C9—C10	1.498 (9)	O1W—H1WA	0.8500
C10—C14	1.382 (10)	O1W—H1WB	0.8510
C10—C11	1.409 (9)	O2W—H2WA	0.8500
C11—C12	1.370 (10)	O2W—H2WB	0.8520
C11—H11	0.9300	O3W—H3WA	0.8503
C12—N2	1.331 (10)	O3W—H3WB	0.8522
C12—H12	0.9300	O4W—H4WA	0.8514
C13—N2	1.315 (9)	O4W—H4WB	0.8504
C13—C14	1.385 (10)		
			
O1—Co1—O1^i^	180.0 (2)	C12—C11—H11	120.4
O1—Co1—N4^ii^	92.16 (19)	C10—C11—H11	120.4
O1^i^—Co1—N4^ii^	87.84 (19)	N2—C12—C11	123.6 (7)
O1—Co1—N4^iii^	87.84 (19)	N2—C12—H12	118.2
O1^i^—Co1—N4^iii^	92.16 (19)	C11—C12—H12	118.2
N4^ii^—Co1—N4^iii^	180.0 (3)	N2—C13—C14	124.7 (7)
O1—Co1—O1W^i^	85.97 (19)	N2—C13—H13	117.6
O1^i^—Co1—O1W^i^	94.03 (19)	C14—C13—H13	117.6
N4^ii^—Co1—O1W^i^	89.3 (2)	C10—C14—C13	118.4 (6)
N4^iii^—Co1—O1W^i^	90.7 (2)	C10—C14—H14	120.8
O1—Co1—O1W	94.03 (19)	C13—C14—H14	120.8
O1^i^—Co1—O1W	85.97 (19)	O6—C15—O7	120.1 (5)
N4^ii^—Co1—O1W	90.7 (2)	O6—C15—C16	119.1 (5)
N4^iii^—Co1—O1W	89.3 (2)	O7—C15—C16	120.8 (5)
O1W^i^—Co1—O1W	180.0 (2)	C21—C16—C17	120.8 (6)
O2^iv^—Ho1—O2W	82.48 (17)	C21—C16—C15	116.6 (5)
O2^iv^—Ho1—O9^v^	89.62 (19)	C17—C16—C15	122.6 (5)
O2W—Ho1—O9^v^	138.85 (17)	C18—C17—C16	119.6 (6)
O2^iv^—Ho1—O6	81.57 (16)	C18—C17—H17	120.2
O2W—Ho1—O6	138.55 (17)	C16—C17—H17	120.2
O9^v^—Ho1—O6	78.95 (15)	C17—C18—C19	120.3 (6)
O2^iv^—Ho1—O3	153.22 (17)	C17—C18—C22	123.0 (6)
O2W—Ho1—O3	122.18 (16)	C19—C18—C22	116.7 (5)
O9^v^—Ho1—O3	78.12 (19)	C20—C19—C18	119.9 (6)
O6—Ho1—O3	72.81 (15)	C20—C19—H19	120.1
O2^iv^—Ho1—O4	152.59 (18)	C18—C19—H19	120.1
O2W—Ho1—O4	71.43 (17)	C19—C20—C21	119.7 (5)
O9^v^—Ho1—O4	104.37 (19)	C19—C20—N3	119.3 (5)
O6—Ho1—O4	123.81 (16)	C21—C20—N3	121.0 (5)
O3—Ho1—O4	54.16 (15)	C16—C21—C20	119.6 (6)
O2^iv^—Ho1—O8^v^	84.81 (18)	C16—C21—H21	120.2
O2W—Ho1—O8^v^	84.52 (17)	C20—C21—H21	120.2
O9^v^—Ho1—O8^v^	54.48 (15)	O9—C22—O8	118.7 (6)
O6—Ho1—O8^v^	131.43 (15)	O9—C22—C18	120.9 (5)
O3—Ho1—O8^v^	106.24 (18)	O8—C22—C18	120.4 (5)
O4—Ho1—O8^v^	84.49 (17)	O9—C22—Ho1^vi^	56.6 (3)
O2^iv^—Ho1—O7	86.64 (18)	O8—C22—Ho1^vi^	62.1 (3)
O2W—Ho1—O7	86.51 (17)	C18—C22—Ho1^vi^	176.7 (4)
O9^v^—Ho1—O7	133.46 (15)	O10—C23—N3	123.5 (6)
O6—Ho1—O7	54.60 (14)	O10—C23—C24	120.2 (6)
O3—Ho1—O7	84.87 (18)	N3—C23—C24	116.3 (5)
O4—Ho1—O7	99.70 (18)	C28—C24—C25	118.0 (6)
O8^v^—Ho1—O7	168.33 (16)	C28—C24—C23	124.5 (6)
O2^iv^—Ho1—C22^v^	86.17 (18)	C25—C24—C23	117.5 (6)
O2W—Ho1—C22^v^	112.05 (19)	C26—C25—C24	118.8 (6)
O9^v^—Ho1—C22^v^	26.84 (16)	C26—C25—H25	120.6
O6—Ho1—C22^v^	104.75 (16)	C24—C25—H25	120.6
O3—Ho1—C22^v^	92.79 (19)	N4—C26—C25	123.8 (6)
O4—Ho1—C22^v^	95.75 (18)	N4—C26—H26	118.1
O8^v^—Ho1—C22^v^	27.66 (17)	C25—C26—H26	118.1
O7—Ho1—C22^v^	158.98 (17)	N4—C27—C28	122.8 (6)
C6—C1—C2	120.0 (5)	N4—C27—H27	118.6
C6—C1—C8	120.6 (5)	C28—C27—H27	118.6
C2—C1—C8	119.2 (5)	C27—C28—C24	119.5 (6)
C3—C2—C1	118.7 (6)	C27—C28—H28	120.3
C3—C2—H2	120.6	C24—C28—H28	120.3
C1—C2—H2	120.6	C9—N1—C5	125.3 (5)
C4—C3—C2	121.3 (6)	C9—N1—H1	117.4
C4—C3—C7	118.1 (5)	C5—N1—H1	117.4
C2—C3—C7	120.6 (5)	C13—N2—C12	116.9 (6)
C3—C4—C5	120.0 (6)	C23—N3—C20	120.8 (5)
C3—C4—H4	120.0	C23—N3—H3	119.6
C5—C4—H4	120.0	C20—N3—H3	119.6
C4—C5—C6	119.2 (6)	C26—N4—C27	117.0 (6)
C4—C5—N1	122.4 (5)	C26—N4—Co1^vii^	121.1 (4)
C6—C5—N1	118.4 (5)	C27—N4—Co1^vii^	121.5 (4)
C1—C6—C5	120.7 (6)	C8—O1—Co1	144.4 (4)
C1—C6—H6	119.6	C8—O2—Ho1^viii^	153.1 (4)
C5—C6—H6	119.6	C7—O3—Ho1	94.4 (4)
O3—C7—O4	118.3 (6)	C7—O4—Ho1	93.0 (4)
O3—C7—C3	120.6 (5)	C15—O6—Ho1	95.1 (3)
O4—C7—C3	121.0 (6)	C15—O7—Ho1	90.1 (4)
O1—C8—O2	124.3 (6)	C22—O8—Ho1^vi^	90.2 (4)
O1—C8—C1	119.4 (5)	C22—O9—Ho1^vi^	96.5 (4)
O2—C8—C1	116.3 (5)	Co1—O1W—H1WA	117.2
O5—C9—N1	123.5 (6)	Co1—O1W—H1WB	112.7
O5—C9—C10	119.3 (6)	H1WA—O1W—H1WB	117.3
N1—C9—C10	117.1 (5)	Ho1—O2W—H2WA	113.0
C14—C10—C11	117.2 (6)	Ho1—O2W—H2WB	110.9
C14—C10—C9	125.8 (6)	H2WA—O2W—H2WB	113.5
C11—C10—C9	116.9 (6)	H3WA—O3W—H3WB	108.4
C12—C11—C10	119.1 (7)	H4WA—O4W—H4WB	112.3
			
C6—C1—C2—C3	−0.6 (9)	C6—C5—N1—C9	161.2 (6)
C8—C1—C2—C3	−175.6 (5)	C14—C13—N2—C12	−0.4 (11)
C1—C2—C3—C4	2.4 (9)	C11—C12—N2—C13	−0.3 (12)
C1—C2—C3—C7	179.7 (6)	O10—C23—N3—C20	9.1 (10)
C2—C3—C4—C5	−1.0 (9)	C24—C23—N3—C20	−170.5 (5)
C7—C3—C4—C5	−178.4 (5)	C19—C20—N3—C23	132.3 (6)
C3—C4—C5—C6	−2.1 (9)	C21—C20—N3—C23	−47.7 (8)
C3—C4—C5—N1	179.5 (6)	C25—C26—N4—C27	−1.8 (10)
C2—C1—C6—C5	−2.5 (9)	C25—C26—N4—Co1^vii^	171.2 (5)
C8—C1—C6—C5	172.4 (5)	C28—C27—N4—C26	3.5 (10)
C4—C5—C6—C1	3.9 (9)	C28—C27—N4—Co1^vii^	−169.4 (5)
N1—C5—C6—C1	−177.7 (5)	O2—C8—O1—Co1	−122.1 (7)
C4—C3—C7—O3	16.9 (9)	C1—C8—O1—Co1	58.6 (10)
C2—C3—C7—O3	−160.6 (6)	N4^ii^—Co1—O1—C8	−28.2 (8)
C4—C3—C7—O4	−162.6 (6)	N4^iii^—Co1—O1—C8	151.8 (8)
C2—C3—C7—O4	20.0 (9)	O1W^i^—Co1—O1—C8	60.9 (7)
C6—C1—C8—O1	−148.0 (6)	O1W—Co1—O1—C8	−119.1 (7)
C2—C1—C8—O1	26.9 (8)	O1—C8—O2—Ho1^viii^	69.4 (12)
C6—C1—C8—O2	32.6 (8)	C1—C8—O2—Ho1^viii^	−111.3 (9)
C2—C1—C8—O2	−152.5 (6)	O4—C7—O3—Ho1	3.2 (7)
O5—C9—C10—C14	−165.1 (7)	C3—C7—O3—Ho1	−176.3 (5)
N1—C9—C10—C14	18.0 (10)	O2^iv^—Ho1—O3—C7	−179.7 (4)
O5—C9—C10—C11	10.0 (10)	O2W—Ho1—O3—C7	−25.1 (5)
N1—C9—C10—C11	−166.8 (6)	O9^v^—Ho1—O3—C7	115.9 (4)
C14—C10—C11—C12	−0.2 (10)	O6—Ho1—O3—C7	−162.2 (4)
C9—C10—C11—C12	−175.8 (7)	O4—Ho1—O3—C7	−1.8 (4)
C10—C11—C12—N2	0.6 (12)	O8^v^—Ho1—O3—C7	68.7 (4)
C11—C10—C14—C13	−0.4 (10)	O7—Ho1—O3—C7	−107.7 (4)
C9—C10—C14—C13	174.7 (6)	C22^v^—Ho1—O3—C7	93.3 (4)
N2—C13—C14—C10	0.7 (11)	O3—C7—O4—Ho1	−3.1 (7)
O6—C15—C16—C21	−4.9 (9)	C3—C7—O4—Ho1	176.3 (5)
O7—C15—C16—C21	174.4 (6)	O2^iv^—Ho1—O4—C7	179.8 (4)
O6—C15—C16—C17	176.2 (6)	O2W—Ho1—O4—C7	161.2 (5)
O7—C15—C16—C17	−4.6 (9)	O9^v^—Ho1—O4—C7	−61.6 (4)
C21—C16—C17—C18	2.2 (9)	O6—Ho1—O4—C7	24.6 (5)
C15—C16—C17—C18	−179.0 (6)	O3—Ho1—O4—C7	1.8 (4)
C16—C17—C18—C19	−1.3 (9)	O8^v^—Ho1—O4—C7	−112.8 (4)
C16—C17—C18—C22	180.0 (6)	O7—Ho1—O4—C7	78.2 (4)
C17—C18—C19—C20	−1.6 (9)	C22^v^—Ho1—O4—C7	−87.5 (4)
C22—C18—C19—C20	177.2 (6)	O7—C15—O6—Ho1	4.0 (6)
C18—C19—C20—C21	3.5 (9)	C16—C15—O6—Ho1	−176.8 (5)
C18—C19—C20—N3	−176.5 (6)	O2^iv^—Ho1—O6—C15	−94.1 (4)
C17—C16—C21—C20	−0.2 (9)	O2W—Ho1—O6—C15	−25.7 (5)
C15—C16—C21—C20	−179.2 (5)	O9^v^—Ho1—O6—C15	174.6 (4)
C19—C20—C21—C16	−2.6 (9)	O3—Ho1—O6—C15	93.8 (4)
N3—C20—C21—C16	177.4 (6)	O4—Ho1—O6—C15	74.6 (4)
C17—C18—C22—O9	−167.3 (6)	O8^v^—Ho1—O6—C15	−169.5 (3)
C19—C18—C22—O9	13.9 (9)	O7—Ho1—O6—C15	−2.2 (3)
C17—C18—C22—O8	12.4 (9)	C22^v^—Ho1—O6—C15	−177.9 (4)
C19—C18—C22—O8	−166.4 (6)	O6—C15—O7—Ho1	−3.8 (6)
O10—C23—C24—C28	133.8 (7)	C16—C15—O7—Ho1	176.9 (5)
N3—C23—C24—C28	−46.7 (9)	O2^iv^—Ho1—O7—C15	84.2 (4)
O10—C23—C24—C25	−42.3 (9)	O2W—Ho1—O7—C15	166.9 (4)
N3—C23—C24—C25	137.2 (6)	O9^v^—Ho1—O7—C15	−2.1 (5)
C28—C24—C25—C26	2.3 (9)	O6—Ho1—O7—C15	2.2 (3)
C23—C24—C25—C26	178.6 (6)	O3—Ho1—O7—C15	−70.4 (4)
C24—C25—C26—N4	−1.1 (10)	O4—Ho1—O7—C15	−122.7 (4)
N4—C27—C28—C24	−2.4 (10)	O8^v^—Ho1—O7—C15	127.1 (7)
C25—C24—C28—C27	−0.6 (10)	C22^v^—Ho1—O7—C15	14.0 (7)
C23—C24—C28—C27	−176.7 (6)	O9—C22—O8—Ho1^vi^	−2.7 (6)
O5—C9—N1—C5	6.6 (11)	C18—C22—O8—Ho1^vi^	177.6 (5)
C10—C9—N1—C5	−176.7 (6)	O8—C22—O9—Ho1^vi^	2.9 (6)
C4—C5—N1—C9	−20.3 (9)	C18—C22—O9—Ho1^vi^	−177.4 (5)

Symmetry codes: (i) −*x*+2, −*y*+1, −*z*; (ii) −*x*+2, −*y*+2, −*z*+1; (iii) *x*, *y*−1, *z*−1; (iv) *x*, *y*+1, *z*; (v) *x*−1, *y*, *z*; (vi) *x*+1, *y*, *z*; (vii) *x*, *y*+1, *z*+1; (viii) *x*, *y*−1, *z*.

### Hydrogen-bond geometry (Å, °)

**Table:**

*D*—H···*A*	*D*—H	H···*A*	*D*···*A*	*D*—H···*A*
O1W—H1WA···O4W^ix^	0.85	2.00	2.761 (8)	149
O1W—H1WB···O7^x^	0.85	2.26	2.988 (7)	144
O2W—H2WB···N2^xi^	0.85	1.96	2.699 (8)	145
O3W—H3WA···O6^viii^	0.85	2.20	3.048 (8)	177
O3W—H3WB···O9^xii^	0.85	2.20	3.054 (8)	177
O4W—H4WA···O4^ix^	0.85	1.90	2.732 (8)	164
O4W—H4WB···O8^xiii^	0.85	1.94	2.752 (8)	160
N1—H1···O3W	0.86	2.16	2.996 (7)	165
N3—H3···O3^ii^	0.86	2.16	2.933 (7)	150

Symmetry codes: (ix) −*x*+1, −*y*+1, −*z*; (x) −*x*+2, −*y*+2, −*z*; (xi) *x*, *y*+1, *z*−1; (viii) *x*, *y*−1, *z*; (xii) −*x*+2, −*y*+1, −*z*+1; (xiii) *x*−1, *y*−1, *z*; (ii) −*x*+2, −*y*+2, −*z*+1.
